# Chemokines in patients with Alzheimer's disease: A meta-analysis

**DOI:** 10.3389/fnagi.2023.1047810

**Published:** 2023-03-09

**Authors:** Hecheng Wang, Yu Zong, Lei Zhu, Weiyi Wang, Yanshuo Han

**Affiliations:** ^1^School of Life and Pharmaceutical Sciences, Dalian University of Technology, Panjin, China; ^2^Department of Cardiovascular Diseases, Civil Aviation General Hospital, Peking University, Beijing, China

**Keywords:** Alzheimer's disease, chemokines, meta-analysis, plasma, CSF

## Abstract

**Background:**

Alzheimer's disease (AD) is the most common neurodegenerative disease in elderly people. Many researches have reported that neuroinflammation is related to AD. Chemokines are a class of small cytokines that play important roles in cell migration and cell communication, which involved in neuroinflammation. Up to now there is no meta-analysis to explore the difference of chemokines between AD patients and healthy elderly individuals.

**Method:**

We searched PubMed, Web of science, Cochrane library, EMBASE and Scopus databases from inception to January 2022. Data were extracted by two independent reviewers, and the Review Manager 5.3 was used for the meta-analysis.

**Result:**

Thirty-two articles were included and analyzed. The total number of participants in the included study was 3,331. We found that the levels of CCL5 (SMD = 2.56, 95% CI: 1.91–3.21), CCL15 (SMD = 3.30, 95% CI: 1.48–5.13) and IP-10 (SMD = 3.88, 95% CI: 1.84–5.91) in the plasma of AD patients were higher than healthy people. MCP-1 protein (SMD = 0.67, 95% CI: 0.29–1.05) in the AD patients' CSF was higher than healthy controls.

**Conclusion:**

These results suggested that chemokines may play an important role in AD. These findings could provide evidences for the diagnosis and treatment of AD.

**Systematic review registration:**

https://www.crd.york.ac.uk/prospero/display_record.php?ID=CRD42021278736, identifier: CRD42021278736.

## 1. Introduction

It is estimated that more than 45 million people worldwide suffer from dementia, and the incidence is expected to triple by 2050 as the life expectancy increasing. Up to now, Alzheimer's disease (AD) is the most common type of dementia, accounting for almost 80% of dementia (Crous-Bou et al., 2017). In clinical practice, AD is mainly diagnosed by testing biomarkers including amyloid β-protein (Aβ) and phosphorylated tau (P-tau) (Scheltens et al., 2021). The detection of biomarkers in AD patients is mainly in the plasma and cerebrospinal fluid (CSF). Aβ_1 − 42_, total tau (T-tau), and p-tau in CSF are the keys to reflect AD pathophysiology (Blennow and Zetterberg, 2018). However, the pathological mechanism of AD is still not clear up to now. Therefore, the discovery of the new biomarker is important to the accurate diagnosis and treatment of AD. Recently, it has been shown that neuroinflammation play an important role in AD and is associated with high levels of inflammatory cytokines (Calsolaro and Edison, 2016; Ramirez et al., 2017; Weng et al., 2022). The CSF and plasma inflammatory cytokine levels in AD patients have attracted great interest in recent years (Chen et al., 2018).

Chemokines are low molecular weight chemotactic cytokines secreted by cells which are involved in neuroinflammation, and related to AD (Vilgelm and Richmond, 2019). They are divided into four families based on structure: CXC, CC, CX3C, and C chemokines. Chemokines exert intracellular signaling by binding to seven-transmembrane G protein-coupled receptors (Griffith et al., 2014; Nagarsheth et al., 2017). Most of the research on chemokines is related to cancer. In recent years, it has been found that chemokines are also involved in neuroinflammation, neuroendocrine, brain development, and thus play functional roles in the central nervous system (Reaux-Le Goazigo et al., 2013; Ghosh et al., 2021). In AD, chemokines play an important role in the development of Aβ plaques and neurofibrillary tangles (Martin and Delarasse, 2018). For example, chemokines and their receptors can promote AD pathology by inducing the production of Aβ (such as CXCR2 and CCR3), and also reduce the deposition of Aβ (such as CX3CR1, CXCR3, and CCR2), respectively. In addition, some chemokines (e.g., MCP−1) and receptors are also involved in tau phosphorylation (e.g., CX3CR1 and CCR3) (El Khoury et al., 2007; Bhaskar et al., 2010; Bakshi et al., 2011; Krauthausen et al., 2015; Zhu et al., 2017; Joly-Amado et al., 2020). These studies have shown that chemokines are closely related to AD. Chemokine levels in AD patients can be detected in the plasma and CSF. However, up to now there is no meta-analysis on chemokines between AD patients and healthy individuals.

In the present work, we aimed to investigate the changes of chemokine between AD patients and healthy controls. We reviewed the published articles, and analyzed the content of chemokines in the plasma and CSF of AD patients and healthy controls. Through the meta-analysis, we found that IP-10, CCL5 and CCL15 of the AD patients' plasma, and MCP-1 in the AD patients' CSF were higher than those of the control group. It could provide the potential biomarkers for AD diagnosis and treatment.

## 2. Methods

### 2.1. Search strategy

We searched PubMed (1995 to January 2022), EMBASE (1995 to January 2022), Web of science (1995 to January 2022), Scopus (1995 to January 2022), and Cochrane Library (1995 to January 2022) with the terms or medical subject terms (MeSH) of “Alzheimer's disease” and “Chemokines.” In order to avoid missing relevant studies from the database, all search results have been included.

### 2.2. Inclusion and exclusion criteria

The identified articles were reviewed carefully in order to qualify for this meta-analysis. All included trials met the following criteria: (1) articles were written in English; (2) participants in the studies were middle-aged and elderly people; (3) according to the fourth edition of the Mental Disorders Diagnostics and Statistics Manual (DSM-IV) and the National Association of Nervous and Communicative Disorders and Stroke/Alzheimer's Disease Institute of Standards (NINCDSADRDA), patients with AD and suspected AD were included (McKhann et al., 1984; Zulauf Logoz, 2014); (4) Studies had been published online by January 2022.

The criteria for the excluded studies were as follows: (1) systematic review or meta-analysis; (2) duplicate study; (3) animal or cell experiment; (4) review or letter; (5) without the full-text; (6) the samples were from brain tissues; (7) there were drug interventions in the process of the clinical trials.

### 2.3. Date extraction

Articles were screened according to inclusion and exclusion criteria. The following data were extracted from each article: first author name, country, publication year, number, gender, age, chemokines, and baseline MMSE. Meanwhile, we assessed the quality of included studies using the Newcastle-Ottawa Quality Assessment Scale (NOS) (Stang, 2010). Due to the inability of all chemokines involved in the current studies, we only analyzed the chemokines which are involved in the included studies in the present work.

### 2.4. Statistical analysis

This meta-analysis was performed using the Review Manager 5.3 software. We separated the sample sources into blood samples and CSF. The content of each chemokine was expressed in the form of mean ± standard deviation (SD). The correlation between chemokine content and AD was assessed by standardized mean difference (SMD) with 95% confidence interval (CI). Heterogeneity was assessed by using *I*^2^ tests and Q tests (Knopman et al., 2001; Higgins and Thompson, 2002; Zintzaras and Ioannidis, 2005). When the heterogeneity is large (*I*^2^ ≥ 50%, *Q*-test: *p* < 0.1), the random effect model is selected. Otherwise, the fixed effects model is selected (*I*^2^ ≤ 50%, *Q*-test: *p* > 0.1). In addition, publication bias was verified by funnel plots. When the funnel plot showed a symmetrical inverted triangle, the included articles were considered free of publication bias.

## 3. Results

### 3.1. Literature search findings

**Figure 1:**
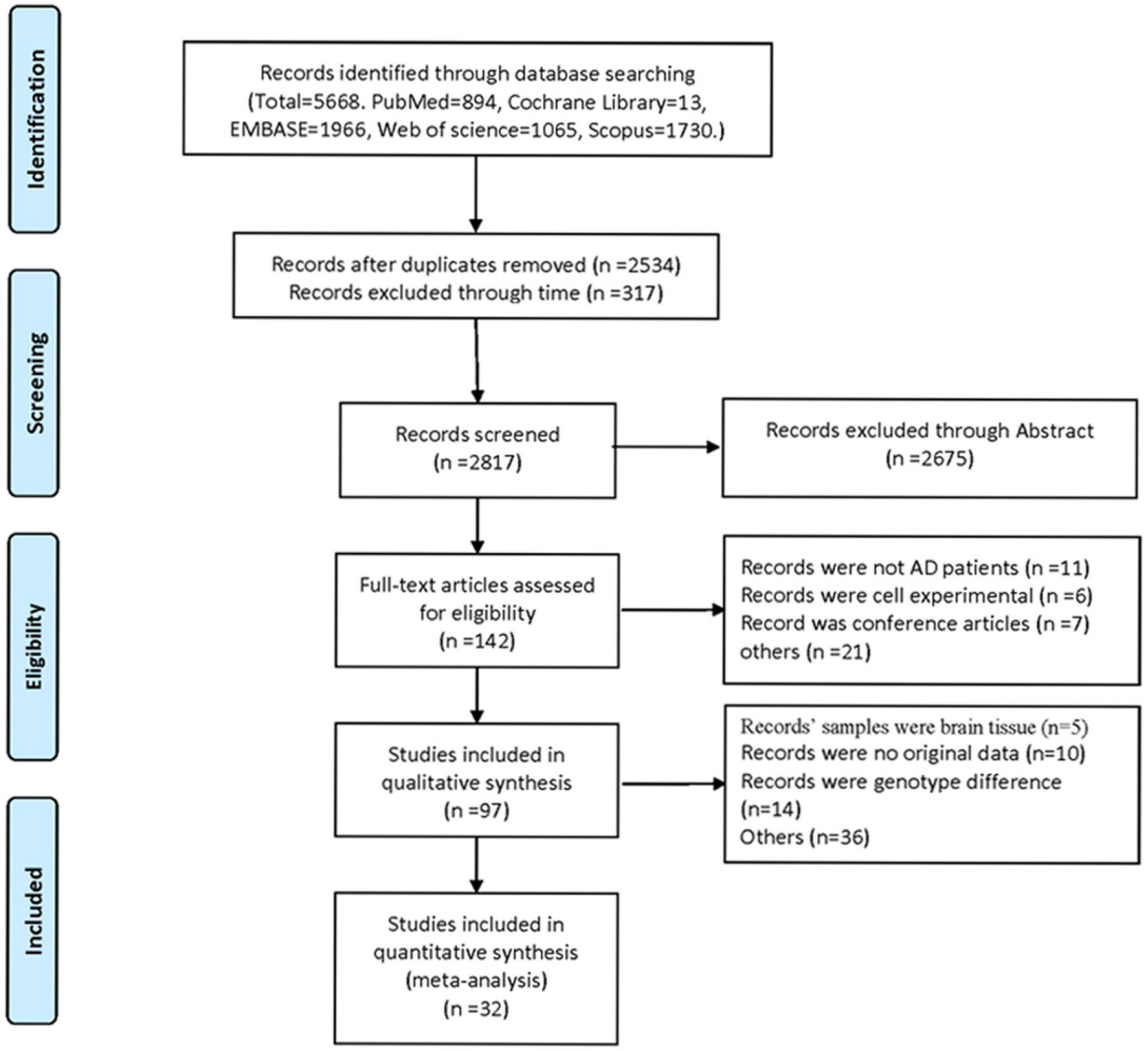
Flowchart of meta-analysis retrieval literature screening.

Five thousand six hundred sixty-eight studies were selected by the literature search, of which 57 papers were considered likely to meet the inclusion criteria after reviewing titles and abstracts. After further reading the full article, 32 studies were finally included in the meta-analysis (Figure 1). These studies were published from 2006 to 2020. The total number of participants in the included study was 3,331. In all studies, the control group referred to healthy individuals and the experimental group referred to AD patients.

### 3.2. Characteristics of the eligible studies

Characteristics of the included studies are shown in Table 1. Depending on the source of chemokines in the included studies, following analyses were also performed in both blood and CSF fractions. Seventeen studies detected chemokines from CSF and 23 studies detected chemokines from blood samples. In addition, there are eight articles detected chemokines in CSF and blood samples at the same time. The quality assessment was based on the Newcastle-Ottawa scale. NOS scores were shown in Table 1, and the quality scores of the included studies were all above five points.

**Table 1:** Baseline characteristics of the included studies.

**Number**	**Author**	**Country**	**Year**	***N* (HC/AD)**	**Gender (% male)**	**Age (HC/AD)**	**Chemokines**	**Baseline MMSE (HC/AD)**	**NOS**
1	Daniela Galimberti	Italy	2006	HC: 24	HC: 25.92%	HC: 71.5 ± 9.8	MCP-1	HC: 28.8 ± 5.9	8
				MCI: 48	AD: 25.53%	MCI: 74.7 ± 6.8		MCI: 26.7 ± 2.1	
				AD: 94		AD: 79.1 ± 7.0		AD: 18.6 ± 11.9	
2	Daniela Galimberti	Italy	2006	HC: 41	HC: 36.59%	HC: 64.0 ± 29.5	IP-10; MCP-1; IL-8	HC: 28–30	7
				MCI: 38	AD: 36.11%	MCI: 68 ± 10.5		MCI: 26–29	
				AD: 36		AD: 59.4 ± 5.4		AD: 15.5 ± 9	
3	Imrich Blasko	Austria	2006	HC: 27	HC: 48.15%	HC: 66.9 ± 9.35	MCP-1; MIP-1α; TNF-α; TGF-β1	HC: 28.8 ± 2.6	7
				AD: 23	AD: 34.78%	AD: 71.9 ± 9.59		AD: 14.8 ± 7.67	
4	Chulhee Choi	Korea	2008	HC: 13	HC: 38.46%	HC: 68.5 ± 7.2	IL-8; IP-10; MCP-1; MIP-1α; TNF-α	HC: n.d.	7
				AD: 11	AD: 18.18%	AD: 73.5 ± 4		AD: n.d.	
5	Christoph Laske	Germany	2008	HC: 30	HC: 66.67%	HC: 69.9 ± 11.1	CXCL12	HC: 28.4 ± 1.6	6
				AD: 30	AD: 40%	AD: 70.5 ± 8.2		AD: 23.6 ± 1.6	
6	Jing Zhang	USA	2008	HC: 95	HC: 46%	HC: 63 ± 12	IL-8	HC: n.d.	8
				AD: 48	AD: 60%	AD: 70 ± 9		AD: n.d.	
7	Josef Marksteiner	Austria	2009	HC: 19	n.d.	HC: 71 ± 5.67	IL1α; IL3; IL8; IL11; MCP3; TNFα	HC: 28.4 ± 1.31	8
				MCI: 44		MCI: 73.5 ± 8.0		MCI: 27.1 ± 1.33	
				AD: 96		AD: 77.0 ± 7.84		AD: 18.8 ± 5.88	
8	Anita Malgorzata Geppert	Poland	2010	HC: 19	HC: 33.33%	HC: 63 ± 12	CCL3	HC: n.d.	7
				AD: 22	AD: 59.09%	AD: 70 ± 9		AD: n.d.	
9	Jôice Dias Corrêa	Brazil	2011	HC: 27	HC: 55.56%	HC: 64.4 ± 11.1	CCL2	HC: n.d.	8
				AD: 22	AD: 22.73%	AD: 74.7 ± 10.2		AD: n.d.	
10	Massimiliano M. Corsi	UK	2011	HC: 6	HC: 33.33%	HC: 73.4 ± 1.1	IL-8; IFN-γ; MCP-1; VEGF	HC: n.d.	8
				AD: 70	AD: 38.89%	AD: 75.6 ± 7.2		AD: n.d.	
11	Tanja Hochstrasser	Austrila	2011	HC: 40	HC: 47.5%	HC: 72.2 ± 6.3	CCL15; CXCL9; CCL3; CCL4; CCL2; CCL22	HC: 28.5 ± 1.3	9
				MCI: 67	MCI: 34.33%	MCI: 73.8 ± 8.0		MCI: 26.9 ± 2.0	
				AD: 92	AD: 22.83%	AD: 78.8 ± 7.1		AD: 18.2 ± 6.1	
12	Niklas Mattsson	Sweden	2011	HC: 19	HC: 47.37%	HC: 74 ± 5	CCL2; IL-6; IL-8	HC: n.d.	8
				AD: 25	AD: 44%	AD: 74 ± 4		AD: n.d.	
13	Karin Westin	Sweden	2012	HC: 30	HC: 43.33%	HC: 72 ± 8	CCL2; CCL11; CCL13; CCL16	HC: 29.3 ± 1.0	7
				MCI: 52	MCI: 53.85%	MCI: 64 ± 9		MCI: 27.3 ± 1.8	
				AD: 47	AD: 23.4% other:65%	AD: 74 ± 6		AD: 26.7 ± 1.4	
				Other: 20		Other: 72 ± 9		Other: 27.0 ± 1.6	
14	Maria Bjö rkqvist	Sweden	2012	HC: 174	HC: 32.76%	HC: 74 ± 9.25	CCL18; CCL5; IL-1α; IL-3; CXCL8; CCL7; CCL15	HC: 29 ± 0.1	8
				AD: 142	AD: 71.83%	AD: 76 ± 7.75		AD: 21 ± 0.4	
15	Mohamad A. Alsadany	Egypt	2012	HC: 25	HC: 48%	HC: 72.8 ± 4.1	IL-8	HC: 27.8 ± 4.4	7
				AD: 25	AD: 44%	AD: 72.2 ± 5.9		AD: 12.56 ± 5.8	
16	M. Reale	Italy	2012	HC: 39	HC: 46.2%	HC: 72.7 ± 4.8	MCP-1; RANTES	HC: 25.7 ± 3.2	8
				AD: 38	AD: 52.6%	AD: 73.8 ± 5.5		AD: 19.2 ± 3.9	
17	Rongzhen Zhang	USA	2013	HC: 31	HC: 51.61%	HC: 75.4 ± 9.5	MCP-1	HC: n.d.	8
				AD: 41	AD: 41.46%	AD: 77.9 ± 7.7		AD: 24.5 ± 2.1	
18	Katharina Stoeck	Germany	2014	HC: 12	HC: 50%	HC: 62.5 ± 2.5	IL-6; IL-7; IL-13; IL-8; MCP-1; TNF-α	HC: n.d.	7
				AD: 35	AD: 42.86%	AD: 69.5 ± 2.5		AD: n.d.	
19	Constance Delaby	France	2015	HC: 24	HC: 46.67%	HC: 66.63 ± 13.3	FABP-3; IL-3; IL-8; MCP-1; MIP-1β; IP-3β; RANTES	HC: 22.9 ± 5.8	8
				AD: 31	AD: 76.65%	AD: 70.84 ± 8.71		MCI: 19.25 ± 5.9	
								AD: 21.8 ± 5.8	
20	Brianne M. Bettcher	USA	2016	HC: 22	HC: 50%	HC: 75.6 ± 7.1	MCP-1; IP-10; MDC; TARC	HC: 29.2 ± 1.1	8
				AD: 149	AD: 45%	AD: 67.8 ± 10.4		AD: 22.4 ± 5.4	
21	Dayana Sazereen Mohd Hasni	Japan	2016	HC: 39	HC: 61.54%	HC: 72.1 ± 5.04	CXCL-1; IL-8; CXCL10; MCP-1; MIP-1α; IL-13; IL-1β; IL-6; IL-12; IFN-γ; TNF-α; IL-10	HC: 30	7
				AD: 39	AD: 43.59%	AD: 80.7 ± 6.41		AD: 19	
22	Raphael Hesse	Germany	2016	HC: 53	HC: 43.48%	HC: 69.5 ± 1.7	IL-1β; IL-8; TNFα	HC: 29 ± 0.49	7
				AD: 45	AD: 29.27%	AD: 67.1 ± 2.4		AD: 21.3 ± 1.7	
23	Julius Popp	Switzerland	2017	HC: 78	HC: 32.05%	HC: 68.4 ± 8.3	BFGF; IFN-γ; IL-15; IL-16; IL-6; IL-7; IL-8; IP-10; MCP-1; MIP-1α; TNFα	HC: 27.8 ± 2.3	8
				AD: 42	AD: 42.86%	AD: 71.4 ± 5.6		AD: 25.2 ± 3.7	
24	Eleanor King	UK	2017	HC: 20	HC: 80%	HC: 75.9 ± 7.3	IL-10; IL-1β; IL-2; IL-4; IL-6; IL-8; TNF-α	HC: 29.1 ± 0.9	8
				MCI: 21	MCI: 33.33%	MCI: 78.5 ± 6.4		MCI: 26.5 ± 2.1	
				AD: 20	AD: 75%	AD: 75.9 ± 6.7		AD: 20.3 ± 4.7	
25	Per Johansson	Sweden	2017	HC: 18	HC: 55.56%	HC: 76 ± 1.75	IL-6; IL-8; IP-10; MCP-1; MIP-1β	HC: 29 ± 0.5	7
				AD: 52	AD: 50%	AD: 74 ± 1.5		AD: 23 ± 1.5	
26	Juan R. Perea	Spain	2018	HC: 14	HC: 57.14%	HC: 64 ± 2.92	CX3CL1	HC: 29 ± 0.88	7
				MCI: 14	MCI: 35.71%	MCI: 70 ± 3.52		MCI: 26 ± 2.95	
				AD: 14	AD: 35.71%	AD: 68 ± 4.18		AD: 21.5 ± 3.5	
27	Carola G Schipke	Germany	2019	HC: 79	HC: 64.56%	HC: 64.5 ± 2.7	BDNF; IGF-1; VEGF; TGF-β1; MCP-1; IL-18	HC: n.d.	8
				AD: 81	AD: 33.33%	AD: 84.9 ± 7.8		AD: 18.0 ± 4.3	
28	Virginia Boccardi	Italy	2019	HC: 87	HC: 40.23%	HC: 75.9 ± 9.0	IFN-α2; TNFα; IL-1α	HC: 28.3 ± 1.7	7
				MCI: 73	MCI: 38.36%	MCI: 77.5 ± 6.3		MCI: 22.1 ± 3.9	
				AD: 129	AD: 28.68%	AD: 81.0 ± 6.2		AD: 16.2 ± 5.0	
29	Whitney Wharton	USA	2019	HC: 51	HC: 41.18%	HC: 69.5 ± 7.1	IL-8; IP-10	HC: n.d.	7
				AD: 74	AD: 45.95%	AD: 69.9 ± 7.5		AD: n.d.	
30	Fernando Gongora-Rivera	Mexico	2019	HC: 49	HC: 24.5%	HC: 72.85 ± 6.59	MIF; SDG-1α	HC: 27.87 ± 2.19	7
				AD: 29	AD: 17.2%	AD: 75.34 ± 5.3		AD: 14.44 ± 5.6	
31	Tugba Ozturk	USA	2019	HC: 44	HC: 45%	HC: 71.1 ± 6.4	IL-8; IP-10; CX3CL1; TNF-α; IL-10	HC: n.d.	8
				AD: 25	AD: 48%	AD: 72.0 ± 11.1		AD: n.d.	
32	Agnieszka Kulczyńska-Przybik	Poland	2020	HC: 20	n.d.	HC: n.d.	CX3CL1; CCL-2; YKL-40	HC: n.d.	7
				MCI: 18		AD: n.d.		AD: n.d.	
				AD: 42					

MMSE, Mini-mental State Examination; NOS, Newcastle-Ottawa Quality Assessment Scale; AD, Alzheimer's disease; HC, healthy controls; MCI, mild cognitive impairment; MCP, monocyte chemoattractant protein; IP-10 IFN-γ-inducible protein 10; IL, interleukin; MIP, macrophage inflammatory proteins; TNF, tumor necrosis factor; CCL, CC chemokine ligand; TGF, transforming growth factor; CXCL, CXC chemokine ligand; IFN, interferon; RANTES, regulated on activation, normal T-expressed, and presumably secreted; FABP fatty acid binding protein; BFGF, basic fibroblast growth factor; BDNF, brain-derived neurotrophic factor; IGF-1, insulin-like growth factor 1; MIF, macrophage migration inhibitory factor; SDG, stromal derived growth factor; VEGF, Vascular endothelial growth factor; YKL-40, Chitinase-3-like protein 1.

### 3.3. Chemokines in the plasma

**Figure 2:**
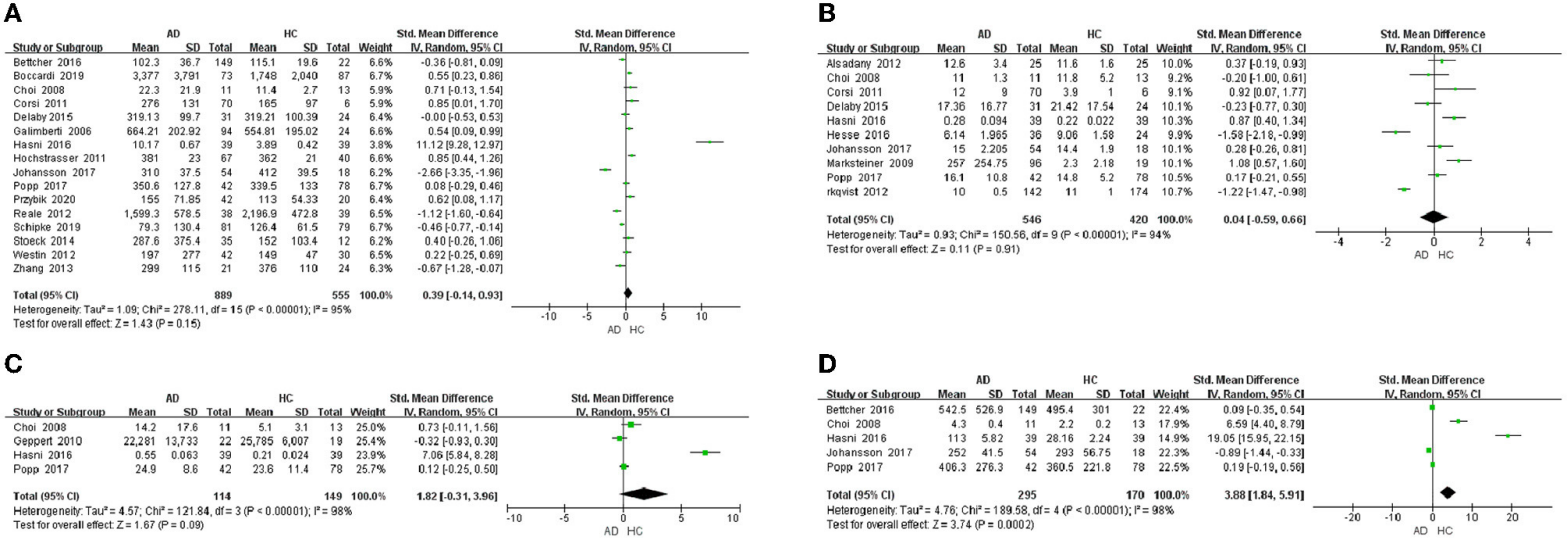
Chemokine levels in the plasma of AD patients. **(A)** MCP-1 protein content, **(B)** IL-8 protein content, **(C)** MIP-1α protein content, and **(D)** IP-10 protein content.

This meta-analysis was performed by Review Manager 5.3 software. In the 32 included studies, there were 23 studies including the data about blood samples. For blood samples, a total of 8 chemokines were analyzed. MCP-1, IL-8, MIP-1α, IP-10, MIP-1β, SDF-1α, CCL5, and CCL15 were included in the meta-analysis, respectively. Our meta-analysis showed that MCP-1 protein level (SMD = 0.39, 95% CI: −0.14 to 0.93), IL-8 protein level (SMD = 0.04, 95% CI: −0.59 to 0.66) and MIP-1α protein level (SMD = 1.82, 95% CI: −0.31 to 3.96) were not significantly different in blood samples between AD patients and healthy individuals (Figures 2A–C, respectively). The levels of IP-10 protein (SMD = 3.88, 95% CI: 1.84–5.91) were higher significantly in blood samples from AD patients than in those from healthy individuals (Figure 2D). Funnel plots were performed to detect the potential publication bias for meta-analysis of MCP-1, IL-8, MIP-1α and IP-10 protein level. The forest plots showed that the included studies for each chemokine analysis were distributed symmetrically, with less publication bias. Beyond that, we also analyzed the protein levels of MIP-1β, SDF-1α, CCL5, and CCL15 in the blood of AD patients and healthy people. We found that the levels of CCL5 (SMD = 2.56, 95% CI: 1.91–3.21) and CCL15 (SMD = 3.30, 95% CI: 1.48–5.13) in the plasma of AD patients were higher than those of healthy people, while the levels of MIP-1β (SMD = 0.32, 95% CI: −0.50 to 0.57) and SDF-1α (SMD = 0.03, 95% CI: −1.56 to 1.62) showed no significant difference (Table 2).

**Table 2:** The amount of chemokines in the blood samples between AD patients and healthy controls.

**Chemokines**	**Number**	**Number of participants**		**Effect size**	**Heterogeneity**	
		**AD**	**HC**	**SMD [95% CI]**	*I* ^2^	***p*** **-value**
MIP-1β	3	152	82	0.32 [−0.50, 0.57]	92%	<0.00001
SDF-1α	2	59	79	0.03 [−1.56, 1.62]	95%	<0.00001
CCL5	2	180	213	2.56 [1.91, 3.21]	75%	0.04
CCL15	2	209	214	3.30 [1.48, 5.13]	97%	<0.00001

AD, Alzheimer's Disease; HC, healthy controls; SMD, standardized mean difference; CI, confidence interval; MIP, Macrophage Inflammatory Proteins; SDF, stromal cell-derived factor; CCL, CC chemokine ligand.

### 3.4. Chemokines in the CSF

**Figure 3:**
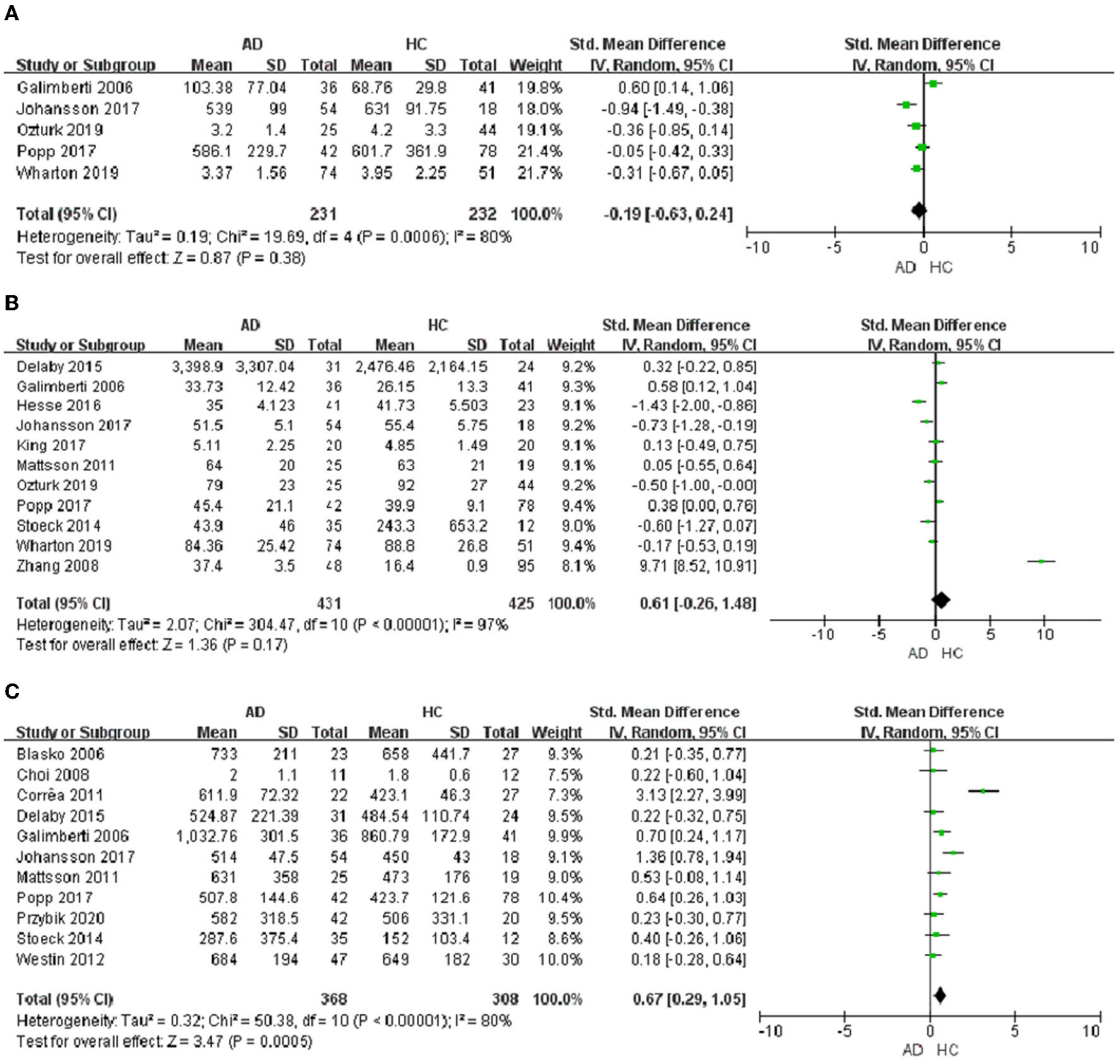
Chemokine levels in the CSF of AD patients. **(A)** IP-10 protein content, **(B)** IL-8 protein content, and **(C)** MCP-1 protein content.

In the 32 included studies, 17 studies were about the chemokines of CSF. In CSF samples, a total of 5 chemokines were analyzed. MCP-1, IL-8, IP-10, MIP-1β, and CX3CL1 were included in the meta-analysis, respectively. The outcomes showed that IP-10 protein level (SMD = −0.19, 95% CI: −0.63 to 0.24) and IL-8 protein level (SMD = 0.61, 95% CI: −0.26 to 1.48) were not significantly different in CSF samples from AD patients and healthy individuals (Figures 3A, B). And then, we found that the level of MCP-1 protein (SMD = 0.67, 95% CI: 0.29–1.05) in AD patients was higher than that in healthy controls (Figure 3C). Meanwhile, we analyzed the protein level of MIP-1β and CX3CL1. The outcomes displayed that MIP-1β (SMD = 0.92, 95% CI: −0.82 to 6.25) and CX3CL1 (SMD = −0.61, 95% CI: −2.11 to 0.89) showed no significant difference between AD patients and healthy controls (Table 3).

**Table 3:** The amount of chemokines in the CSF between AD patients and healthy controls.

**Chemokines**	**Number**	**Number of participants**		**Effect size**	**Heterogeneity**	
		**AD**	**HC**	**SMD [95% CI]**	*I* ^2^	***p*** **-value**
MIP-1β	2	85	42	0.92 [−0.82, 2.65]	95%	<0.0001
CX3CL1	3	81	78	−0.61 [−2.11, 0.89]	94%	<0.0001

AD, Alzheimer's Disease; HC, healthy controls; SMD, standardized mean difference; CI, confidence interval; MIP, Macrophage Inflammatory Proteins; CXCL, CXC chemokine ligand.

**Figure 4:**
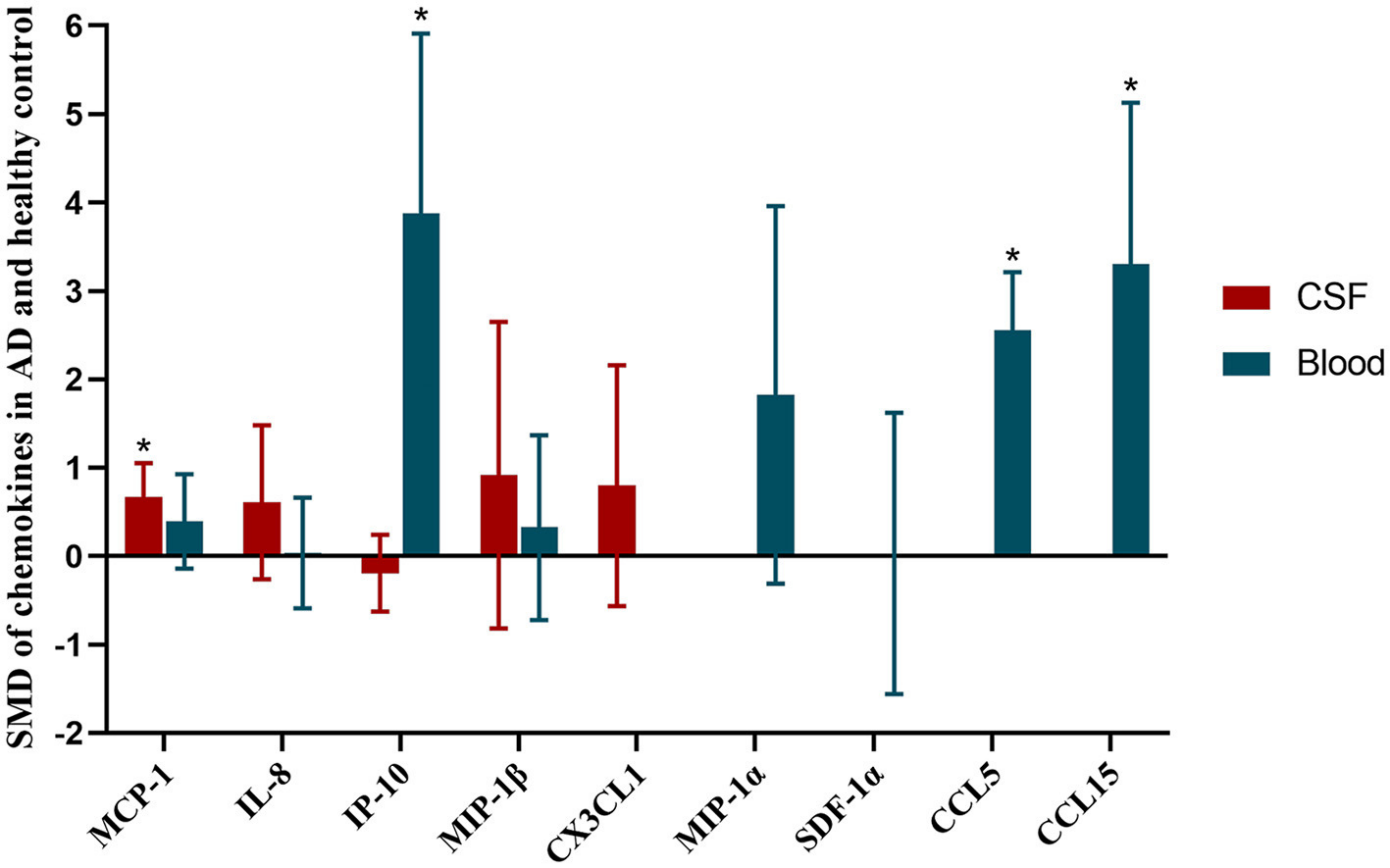
SMD of chemokines in AD and healthy control. Asterisks indicate significant significance between groups. Red histogram shows SMD between AD and healthy controls in CSF samples. Blue histogram shows SMD between AD and healthy controls in blood samples.

Finally, all the SMD of chemokines between AD patients and healthy controls, and their 95% CIs were showed in the Figure 4.

## 4. Discussion

To our knowledge, this is the first systematic review and meta-analysis to summarize the chemokines in the plasma and CSF between AD patients and healthy individuals. The chemokine system is involved in the brain microenvironment of AD patients (Azizi et al., 2014). Chemokines are important regulators of both the central and peripheral immune response, and play an important role in inflammatory processes of the brain. The chemokines in AD patients' plasma and CSF change significantly compared to healthy controls, which may be related to AD (Liu C. et al., 2014). Many studies have found that the chemokines regulate the infiltration of peripheral mononuclear cells into the AD brain, and/or the accumulation of Aβ deposition in microglia cells, and involved in tau phosphorylation (Zhu et al., 2017; Guedes et al., 2018). Therefore, chemokines and their receptors were involved in the occurrence and progression of AD (Liu C. et al., 2014; Wojcieszak et al., 2022). These results combined with the present work suggest that the chemokines may play an important role in AD.

In this meta-analysis, we detected higher protein concentrations of CCL5, IP-10, and CCL15 in the plasma of AD patients than in that of healthy people. Among them, the previous studies have shown that the expression of CCL5 is increased in the brain microenvironment circulation of AD patients (Chen et al., 2004; Sharma et al., 2007; Lumpkins et al., 2008; Tripathy et al., 2010). Li et al. (2023) have reported that the *CCL5* gene is increased in the brain of AD patients and AD mice. In addition, soluble CCL5 could be activated by Aβ, which is related to AD. CCL5 was enhanced with Mn exposure inducing neuroinflammation which contributes to AD (Kirkley et al., 2017). As the receptor of CCL5, the expression of CCR5 is increased on some reactive microglia in AD patients compared to healthy controls (Xia et al., 1998). Furthermore, the IP-10 levels in the plasma of AD patients was up-regulated in the present work. However, IP-10 concentrations of plasma correlating positively with AD was controversial in the previous studies and other meta-analysis (Lai et al., 2017; Su et al., 2019). It was found that IP-10 which was co-localized with Aβ plaques was increased in aged brain of AD mice (Duan et al., 2008; Guedes et al., 2018). As the pro-inflammatory chemokine, IP-10 was up-regulated when mice were injected with Aβ (Lai et al., 2013). Besides, in the plasma, higher level of IP-10 were associated with the increased levels of CSF t-tau and p-tau (Bettcher et al., 2018). Therefore, the changes of IP-10 protein levels may reflect the progression of AD. In clinical trials, compared with the control group, the protein level of CCL15 in monocytes of AD patients was down-regulated significantly (Hochstrasser et al., 2011). As a potential target of AD, neurotrophin-3 has aroused the interest of AD researchers. Meanwhile some studies have found that CCL15 appears in a meta feature with neurotrophin-3 (Rocha De Paula et al., 2011). Therefore, we speculated that CCL15 might be related to AD. In the previous studies, it was reported that CCL15, CCL5, and IP-10 were involved in neuroinflammation (Ubogu et al., 2006; Hochstrasser et al., 2011; Pan et al., 2022). In the collection of clinical samples, blood sample is more readily obtained compared to CSF. The levels of IP-10, CCL5 and CCL15 which have been found to be significantly up-regulated in the plasma of AD patients may be the potential biomarkers for the clinical diagnosis of AD.

In the present work, the performance of the chemokines in the periphery was not the same as in CSF. One of the reasons is heterogeneity of the population and the different methods used in the research. In addition, cerebrospinal fluid is a colorless and transparent fluid existing in the ventricles and subarachnoid space. Owing to the blood-brain barrier, the content and the concentration of the proteins in the blood are quite different from that in the CSF (Stolp and Dziegielewska, 2009). The detection of biomarkers (e.g., Aβ levels) in CSF is important in the diagnosis and treatment of neurodegenerative diseases. For example, detection of Aβ_1 − 42_ and p-tau in CSF is a well-established method in AD diagnosis (Iwatsubo et al., 1994; Motter et al., 1995; Lleo et al., 2015; Olsson et al., 2016). Many studies have shown that chemokines in CSF could impact on tau hyperphosphorylation and the level of Aβ. In our study, it was found that the contents of MCP-1 in the CSF of AD patients was higher than that of healthy controls. MCP-1 (also called CCL2), a chemokine belonging to the CC family, is one of the chemokines for regulating monocyte/macrophage migration and infiltration (Sozzani et al., 1994). In recent years, many studies have found that MCP-1 play important roles in inflammation and related to AD (Chen et al., 2016; Wojcieszak et al., 2022). MCP-1 was expressed by microglia. It was found that MCP-1 could facilitate Aβ oligomer formation and induce neuronal loss by binding to CC-chemokine receptor 2 in pathology and physiology of AD (Britschgi and Wyss-Coray, 2007; Kiyota et al., 2009; Conductier et al., 2010). In the animal experiment, MCP-1 could accelerate Tau pathology together with glia activation (Joly-Amado et al., 2020). MCP-1 in HP resilient cases were up-regulated in AD patients compared to healthy individuals (Barroeta-Espar et al., 2019). In addition, elevated levels of MCP-1 in AD patients have also been found in other studies and the previous meta-analysis, which are consistent with our outcomes (Galimberti et al., 2006; Lee et al., 2018; Shen et al., 2019). CX3CL1/CX3CR1 is also related to AD. CX3CL1 was increased in blood-brain barrier of AD, as a potential biomarker in the early stages of AD (Verite et al., 2018). Meanwhile, CXC3CR1 deficiency increased the Aβ level, and aggravated tau pathology, inducing cognitive decline (Puntambekar et al., 2022). However, the CX3CL1 was not significantly different between AD and healthy controls in the present work. This may be due to the results of the included studies in the meta-analysis, and the differences in methodological inclusion criteria. Therefore, we concluded that MCP-1 protein in the CSF might be the potential biomarker of AD.

Previous studies have shown that MCP-1, IL-8 are elevated in AD patients' peripheral blood compared to healthy controls, which is inconsistent with our results (Lai et al., 2017; Shen et al., 2019; Su et al., 2019). This may be due to the methodological differences in the inclusion criteria, and the results of the new studies which have been published since the other meta-analysis.

The advantage of the meta-analysis was that the present work had included all of the chemokines which are involved in the included studies, to investigate the levels of chemokine in the plasma and CSF between AD patients and healthy individuals. Some studies have shown that these chemokines and receptors play a neuroprotective role in the brain. Overexpression of CCL5 may reduce the neurotoxicity induced by the infection of HIV, which was related to p38-MAPK and PI3K/Akt pathway (Mocchetti et al., 2013; Liu X. et al., 2014; Campbell et al., 2015). As for IP-10, it was slightly neuroprotective at 10 ng/ml when the mixed or pure neuronal cultures were also exposed to NMDA (Bruno et al., 2000). MCP-1/CCR2 was involved in neuronal survival through PI3K/Akt/NF-κB pathway (Yao et al., 2009). In addition, MCP-1 could reduce the extracellular level of glutamate and the apoptotic cell death to against NMDA toxicity *in vitro* (Bruno et al., 2000; Eugenin et al., 2003). However, many studies have found that these chemokines are involved in AD as pro-inflammatory cytokines. Therefore, we speculated that these chemokines may be the potential inflammatory biomarkers of AD. Further studies are needed to investigate the mechanism of these chemokines in the pathological process of AD and these chemokines correlate with the established biomarkers including Aβ and tau.

There are some limitations in our meta-analysis. First, we performed a risk assessment of the included articles using Review Manager 5.3 software, and one article was at risk of “selective publication.” There may be potential publication bias in the results. And then, some chemokines involved a small amount of studies, and the results may be biased due to the small amount of data. Finally, our search is limited to literatures published in English, which may cause some selection bias.

## 5. Conclusion

This meta-analysis showed that the progression of AD may be associated with the elevated concentrations of chemokines. The concentrations of IP-10, CCL5, and CCL15 were significantly increased in the plasma of AD patients. In the CSF of AD patients, we found that MCP-1 protein was significantly increased. The study could provide a new insight into the diagnosis and treatment of AD.

## Data availability statement

The original contributions presented in the study are included in the article/supplementary material, further inquiries can be directed to the corresponding author/s.

## Author contributions

WW, YH, and HW designed the study and wrote the manuscript. HW and YZ searched the databases, extracted the data, and analyzed the data. HW and LZ conducted the quality assessment of the included studies. All authors reviewed and approved the manuscript.
